# Appropriate Soil Heat Treatment Promotes Growth and Disease Suppression of *Panax notoginseng* by Interfering with the Bacterial Community

**DOI:** 10.4014/jmb.2112.12005

**Published:** 2022-02-17

**Authors:** Ying-Bin Li, Zhi-Ping Zhang, Ye Yuan, Hui-Chuan Huang, Xin-Yue Mei, Fen Du, Min Yang, Yi-Xiang Liu, Shu-Sheng Zhu

**Affiliations:** 1 State Key Laboratory for Conservation and Utilization of Bio-Resources in Yunnan, Yunnan Agricultural University, Kunming 650201, P.R. China; 2 Key Laboratory for Agro-Biodiversity and Pest Control (Ministry of Education), College of Plant Protection, Yunnan Agricultural University, Kunming 650201, P.R. China

**Keywords:** Soil heat treatment, microbial diversity, consecutively cultivated soil, *Panax notoginseng*

## Abstract

In our greenhouse experiment, soil heat treatment groups (50, 80, and 121&deg;C) significantly promoted growth and disease suppression of *Panax notoginseng* in consecutively cultivated soil (CCS) samples (*p* < 0.01), and 80&deg;C worked better than 50&deg;C and 121&deg;C (*p* < 0.01). Furthermore, we found that heat treatment at 80&deg;C changes the microbial diversity in CCS, and the inhibition ratios of culturable microorganisms, such as fungi and actinomycetes, were nearly 100%. However, the heat-tolerant bacterial community was preserved. The 16S rRNA gene and internal transcribed spacer (ITS) sequencing analyses indicated that the soil heat treatment had a greater effect on the Chao1 index and Shannon&rsquo;s diversity index of bacteria than fungi, and the relative abundances of Firmicutes and Proteobacteria were significantly higher than without heating (80 and 121&deg;C, *p* < 0.05). Soil probiotic bacteria, such as *Bacillus* (67%), *Sporosarcina* (9%), *Paenibacillus* (6%), *Paenisporosarcina* (6%), and *Cohnella* (4%), remained in the soil after the 80&deg;C and 121&deg;C heat treatments. Although steam increased the relative abundances of most of the heat-tolerant microbes before sowing, richness and diversity gradually recovered to the level of CCS, regardless of fungi or bacteria, after replanting. Thus, we added heat-tolerant microbes (such as *Bacillus*) after steaming, which reduced the relative abundance of pathogens, recruited antagonistic bacteria, and provided a long-term protective effect compared to the steaming and *Bacillus* alone (*p* < 0.05). Taken together, the current study provides novel insight into sustainable agriculture in a consecutively cultivated system.

## Introduction 

Sanqi ginseng [*Panax notoginseng* (Burk.) F. H. Chen], family Araliaceae, is a well-known traditional Chinese medicine with valued pharmacological activities such as hemostatic [1], antioxidant [2], neuroprotective [3], and antitumor effects [4]. *P. notoginseng* was listed in the European Pharmacopoeia in 2016. However, cultivation of *P. notoginseng* is often threatened by negative plant-soil feedback (NPSF), which results in yield losses estimated at 10–40% and more than 70% [5, 6] due to low seed germination, poor seedling growth, and severe disease [7], along with a requirement of at least 10–30 years for successful replanting [8]. Several factors contribute to these problems, including deterioration of the soil physicochemical characteristics, nutrient imbalance, and the accumulation of autotoxicity [7, 9-12]. Previously identified soil-borne pathogens, including *Ilyonectria destructans*, *I. didynum*, *Fusarium solani*, *F. oxysporum*, *F. flocciferum*, *Phoma herbarum*, and *Phytophthora cactorum* also contribute to NPSF [13-15]. Among these pathogens, *Fusarium* spp. and *Ilyonectria* spp. are the most important [16, 17].

Soil steaming is a clean, effective, and more rapid method than chemical fumigants for organic producers [18, 19] as it increases plant growth rates and strengthens resistance against diseases and pests [19, 20]. However, steaming has low selectivity, which may lead to a biological vacuum and consequent rapid recolonization by pathogens [18]. Therefore, soil remediation should be applied after steaming.

Microbes play an integral role in virtually all soil processes, such that microbial abundance, composition, and activity will largely determine the sustainable productivity of agricultural land [21-24]. Soil microbes enhance plant productivity and protect against soil-borne diseases by driving nutrient cycling and transforming organic materials [25]. Previous studies have confirmed that soil conditions must improve before replanting sanqi ginseng, and steaming at 90°C for 15 min significantly increases seed germination and seedling survival rates [19, 20, 26].

In this study we aimed to understand how soil microbes modulate the yield response and disease control effect after a soil heat treatment. Specifically, different soil heat treatments were applied to analyze changes in the soil microbes in a cultivated soil system using 16S rRNA and internal transcribed spacer (ITS) sequencing. In addition, we confirmed the function of key soil microbes.

## Materials and Methods

### Strains

*Fusarium solani* (strains F3 and F5) and *Ilyonectria destructans* DS006 were provided by the Key Laboratory of Agro-Biodiversity and Pest Management of the Education Ministry of China, Yunnan Agricultural University, Kunming.

### Greenhouse Soil Heat Treatment

The effects of a soil heat treatment on growth and disease suppression of sanqi were assessed in a greenhouse located at the Agricultural Experimental Station of Yunnan Agricultural University, Xundian County, Kunming, China (103.286°E, 25.521°N; altitude of 1960 m), where sanqi has been cultivated continuously from 2014 to 2017. The characteristics of consecutively cultivated soil (CCS) were as follows: pH 7.66; electrical conductivity: 1,108 μs/cm; available phosphorus: 125.20 mg/kg; available potassium: 765.47 mg/kg; alkali-hydrolyzable nitrogen: 107.33 mg/kg; organic matter: 31.72 g/kg. CCS was collected and treated for 30 min at 50, 80, and 121°C. Uncultivated pine forest soil (PFS) samples were used as controls, and the characteristics were as follows: pH 5.17; electrical conductivity: 458 μs/cm; available phosphorus: 5.18 mg/kg; available potassium: 6.90 mg/kg; alkali-hydrolyzable nitrogen: 172.38 mg/kg; organic matter: 47.83 g/kg.

### Sanqi Growth Condition

Sanqi seeds were immersed in 1% sodium hypochlorite for 5 min and washed three times with sterile water; 200 seeds were placed in a nursery tank (1.0 m × 0.5 m, with 0.05 m × 0.05 m spacing) with a soil thickness of 0.1 m and one seed per well. Each treatment had four replicates. All nursery tanks were separated by plastic film and arranged according to a completely randomized block design. The greenhouse was shaded with a polyethylene net that allowed 10% light transmission to mimic the natural conditions for sanqi growth [27]. The temperature was controlled at 18–30°C and strict moisture control was implemented. The seedling emergence rate (SER) was recorded when the plant emergence rate in uncultivated pine soil exceeded 50% April 21. The seedling survival rate (SSR) was recorded 2 months after sowing June 21. The incidence rates of sanqi root rot for the different treatments were analyzed statistically according to a previously described method [7].

### Culturable Microorganisms in CCS

After the 30-min soil heat treatment at a series of temperatures (70, 80, 90, 100, 110, and 121°C), the survival of soil fungi, bacteria, and actinomycetes was determined in CCS. First, soil samples were sieved through a 20-mesh screen to remove plant debris. Then, 10 g of each soil sample was added to 90 ml of sterilized water. After 30 min at 180 rpm, the soil suspension was diluted in a series (from 10^-2^ to 10^-4^). A 50 μl aliquot of the solution was plated on Rose Bengal agar for fungi, beef extract peptone for the bacteria, and Gauze’s Medium No. 1 for the actinomycetes [28]. The colony-forming units (CFUs) were counted after a 4–6-day incubation at 28°C. Three independent biological replicates were used for each treatment. The results are expressed as the inhibition ratio (IR, %) compared to the control (CK) without soil heat treatment.

Inhibition ratio (%) = 100 × (CFUs of control − CFUs of treated sample)/CFUs of control

### Sampling, DNA Extraction, and High-Throughput Sequencing

The CCS samples treated at 50, 80, and 121°C were collected before sowing, and untreated samples were collected as the control. The soil samples of the corresponding treatment were collected at harvest, according to a previously described method [12]. Briefly, the soil samples were randomly collected from 20 pots and mixed into three biological replicates for each treatment. All samples were placed in 5 ml centrifuge tubes and stored at −80°C for DNA extraction and high-throughput sequencing.

The total genomic DNA of each soil sample was extracted using the Fast DNA SPIN Kit for Soil (MP Biomedicals, USA) according to the manufacturer’s instructions. The V4–V5 regions of the bacterial 16S rRNA gene and ITS2 region of the fungal ITS were amplified, respectively, with the 515F/907R [29] and ITS2F/ITS2R [30] primer sets using the ABI GeneAmp 9700 PCR thermocycler (ABI, USA) as follows: initial denaturation at 95°C for 3 min, followed by 27 cycles of 95°C for 30 s, 55°C for 30 s, 72°C for 45 s, and 72°C for 10 min. The purified amplicons were sequenced using the Illumina MiSeq platform at Novogene Corp. (China) according to the standard protocol. Analyses of alpha and beta diversity were performed based on the normalized operational taxonomic unit (OTU) abundance values. All sequences of ITS and 16S rRNA genes can be found in the Short Read Archive (SRA) at NCBI (https://trace.ncbi.nlm.nih.gov/Traces/sra) under accession number PRJNA760266.

### Soil Steaming Treatment in the Field

Steam was generated using an oil-fired steaming boiler (Jiangsu Anxin Boiler Co., Ltd., China). Briefly, the soil was covered with a heat retention sheet and the steam was injected underneath the sheet through an injector and protection tunnel, whereby the soil temperature was raised and maintained at ~80°C for 30 min to a depth of 15 cm. The characteristics of the steaming treatment soil (STS) were as follows: pH 7.65; electrical conductivity: 1,432 μs/cm; available phosphorus: 122.64 mg/kg; available potassium: 841.70 mg/kg; alkali-hydrolyzable nitrogen: 125.83 mg/kg; organic matter: 27.29 g/kg.

### Isolation, Identification and Dual Culture

After the CCS had been treated with oil-fired steam in the field, the bacteria that survived were isolated and cultured on beef extract-peptone medium. The isolates were identified by analyzing the 16S rRNA gene sequence, as described previously [12]. Representative isolates were selected and antagonistic activity against *F. solani* (strains F3 and F5) and *I. destructans* DS006 was tested using the dual culture method according to previous descriptions [31].

### *Bacillus* Amendment after Soil Steaming in the Field

A field study was conducted to confirm the control efficiency of STS combined with *Bacillus* M on sanqi replant failure. First, the CCS cultivated with the same crop for over 4 years was treated with the oil-fired steam boiler, and the isolated *Bacillus* M was used as a seed dressing at a dose of 8 g/kg. Untreated CCS and uncultivated PFS were planted as controls (four replicates per treatment). Once the seeds had sprouted, the roots were irrigated with *Bacillus* M every 2 weeks at a dosage of 4.6 ml/m^2^, which was diluted with 5 L of water in advance. SSR were recorded when 80% of the plants germinated in the uncultivated PFS. Biomass was recorded and measured according to a previous method [7].

To evaluate disease severity, the sanqi roots were harvested, washed with water, and examined as described previously (0: no disease; 1: necrotic lesions < 10% of the taproot; 2: approximately 10–20% of the taproot cankered; 3: approximately 20–40% of the taproot cankered; 4: approximately 40–80% of the taproot blackened; 5: >80% of the taproot blackened) [12].

### Statistical Analysis

GraphPad Prism 8.3 software (GraphPad Software Inc., USA) was used for the statistical analysis. Significant differences in seedling survival, the disease index, the biomass, and diversity indices were detected using Fisher’s LSD test and DPS software (UK). The microbial taxa data were analyzed using R software (version 2.15.3; R Foundation for Statistical Computing, Austria). The means of the alpha diversity indices were compared between treatments using Tukey’s honestly significant difference test. Means of the relative abundances of the dominant microbial genera were compared between treatments using Welch’s *t*-test. Alpha diversity indices were correlated using Pearson’s correlation. Relative abundances of fungi and bacteria at the phylum level were determined based on the Bray Curtis distance and weighted UniFrac distance using R software, to visualize community similarity.

## Results

### Appropriate Soil Heat Treatment Is More Conducive to Sanqi Seedling Growth

**Fig. 1:**
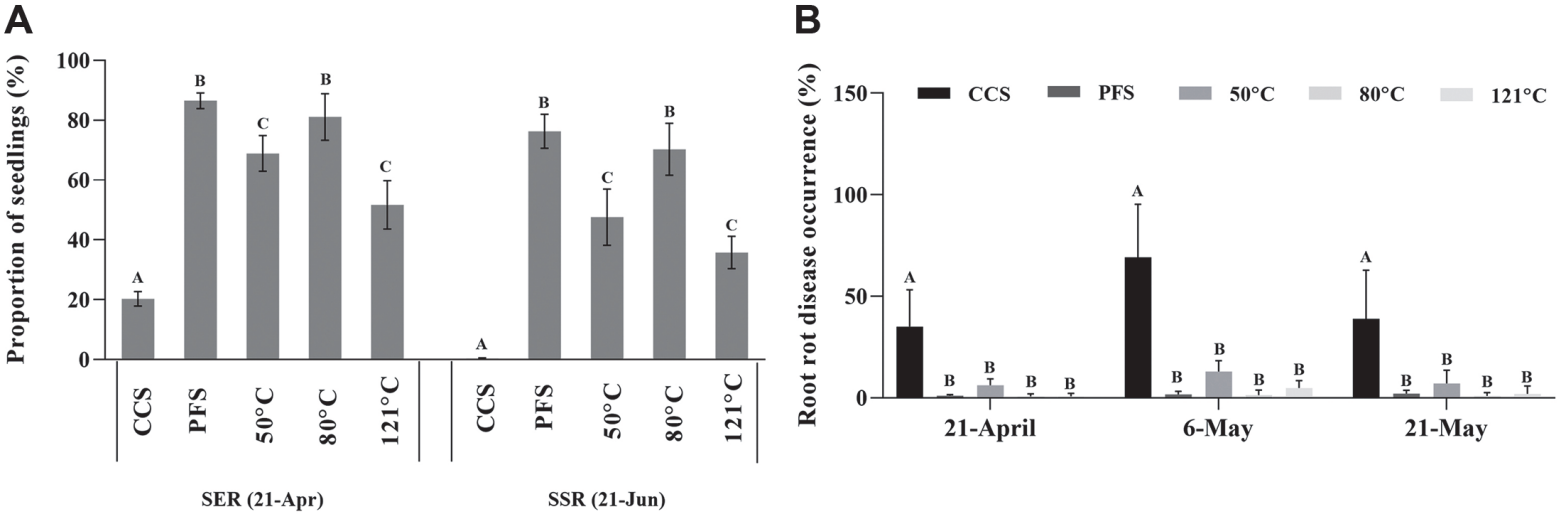
Seedling survival rates on consecutively cultivated soil after heating at 50, 80, and 121°C for 30 min in a greenhouse: (**A**) Seedling survival rates. (**B**) Sanqi root rot disease during the growth stage. The untreated consecutively cultivated soil (CCS) and uncultivated pine forest soil (PFS) were used as controls. *n* = 4 for the control/treatment group. All data are presented as mean ± SD and were compared using Fisher’s least significant difference (LSD) test (*p* < 0.01).

As shown in Fig. 1A, sowing in PFS resulted in the highest SER, of 86.5%. All heat treatment groups maintained higher SERs (*p* < 0.01) of 68.9% at 50°C, 81.1% at 80°C, and 51.7% at 121°C, compared to sowing in CCS. Two months after sowing, the SSR rapidly declined to 0.4% for CCS, whereas the SSR was maintained at 47.6% for 50°C, 70.3% for 80°C, and 35.8% for 121°C (*p* < 0.01). Notably, the SER and SSR were significantly higher when the soil was heated at 80°C compared to those at 50°C and 121°C (*p* < 0.01), which achieved a level equivalent to that of PFS. Additionally, the soil heat treatments significantly reduced disease occurrence compared to CCS (*p* < 0.01)(Fig. 1B). This result indicates that the soil heat treatment helped protect the seedlings, and that 80°C was more conducive for seedling growth on CCS, compared to the high (121°C) and low (50°C) temperature heat treatments.

### Effect of Heat Treatment on Culturable Soil Microorganisms

**Fig. 2:**
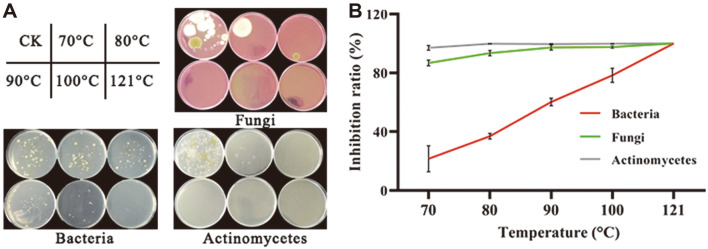
Inhibition ratio (IR) of culturable microorganisms in consecutively cultivated soil after heating at temperatures of 70, 80, 90, 100, and 121°C compared to untreated consecutively cultivated soil (control, CK). (**A**) Isolates grown on different media. (**B**) IR. *n* = 3 for the CK/treatment group. The results are expressed as the IR (%). All data are presented as mean ± SD.

In this study, culturable microorganisms, including fungi, bacteria, and actinomycetes, were counted by colony-counting methods, after CCS was treated at a series of temperatures (Fig. 2A). The IRs were 86.8% and 97.1% for fungi and actinomycetes, respectively, after the 70°C treatment for 30 min. In contrast, the bacteria were heat-tolerant and the IR was 21.7%. Once the temperature reached 80°C, the IRs for fungi and actinomycetes were nearly 100%, whereas the IR for bacteria was 37%. As the temperature was increased in the soil, the number of culturable bacteria gradually fell to zero at 121°C (Fig. 2B). These results suggest that culturable fungi and actinomycetes in soil were more sensitive to heat treatment, and that the heat-tolerant bacterial community may play a crucial role in healthy sanqi growth.

### α-Diversity Analysis of the Bacterial and Fungal Communities after Heat Treatment

**Fig. 3:**
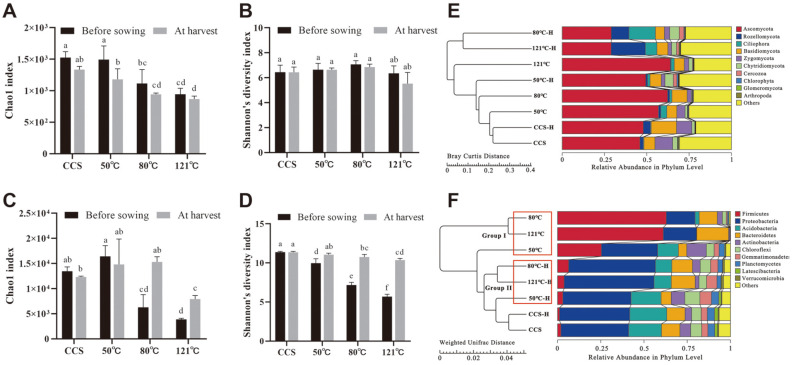
Analysis of α-diversity and phylum-level changes in the fungal and bacterial communities after heating at different growth stages (before sowing and at harvest). (**A, B**) The Chao1 and Shannon’s diversity indices of the fungal community. (**C, D**) The Chao1 and Shannon’s diversity indices of the bacterial community. (**E, F**) The relative abundances of fungi and bacteria at the phylum level, respectively. All data are presented as mean ± standard deviation (SD) and were compared using the least significant difference (LSD) test for α-diversity (*p* < 0.05). “X°C” denotes the phylum level change before sowing after heating at 50, 80, and 121°C for 30 min. “X°C-H” denotes the phylum level change at harvest. Untreated consecutively cultivated soil before sowing (CCS) and at harvest (CCS-H) were used as controls. *n* = 3 for control/treatment group.

To further explore the effect of heat treatment on the changes of microbial diversity in CCS, 16S rRNA and ITS gene amplicon sequencing were performed. The Chao1 index of fungi was significantly lower after heating at 80 and 121°C compared to that of the CCS treatment (before sowing, *p* < 0.05), suggesting that fungi richness decreased with increasing steaming temperature, and the richness index of each treatment at harvest remained at the same level as before sowing (Fig. 3A). The Shannon’s diversity index of heating (50, 80, or 121°C) and replanting (from sowing to harvest) did not affect fungal diversity compared to CCS (*p* < 0.05) (Fig. 3B). The Chao1 and Shannon’s diversity indices for bacteria were significantly lower after heating at 80 and 121°C (before sowing, *p* < 0.05); however, the bacterial richness and diversity of each treatment increased significantly at harvest relative to before sowing, particularly at 80 and 121°C (at harvest, *p* < 0.05) (Figs. 3C and 3D). Taken together, these results indicate that the heat treatments (80 and 121°C) affected the richness of the fungi and bacteria. Nevertheless, replanting was more conducive to recolonization of the bacterial community than the fungal community after soil heat treatment, particularly at 80 and 121°C.

### Phylum Level Changes in the Bacterial and Fungal Communities

The top 10 bacterial and fungal phyla in the community structure were further analyzed. The fungal sequences were predominantly associated with the phyla Ascomycota, Rozellomycota, Ciliophora, and Basidiomycota, and these four phyla accounted for 55–75% of the fungal sequences. The relative abundance of Ascomycota increased before sowing, whereas the relative abundance of Rozellomycota decreased gradually with increasing temperature (50, 80, and 121°C) compared to CCS. However, at harvest, the relative abundance of Ascomycota decreased gradually, and the relative abundance of Rozellomycota increased gradually (50°C-H, 80°C-H, and 121°C-H) compared to CCS-H (Fig. 3E). The bacterial sequences were predominantly associated with the phyla Firmicutes, Proteobacteria, Acidobacteria, and Bacteroidetes, and these four phyla accounted for 70–95% of the bacterial sequences. The relative abundance of Firmicutes before sowing (Group I: 50, 80, and 121°C) was significantly higher than without heating (*p* < 0.05), and the relative abundances of Proteobacteria and Acidobacteria were significantly lower than those in CCS (*p* < 0.05), particularly after heating at 80 and 121°C. At harvest, the relative abundances of the most dominant phyla in the 50°C-H, 80°C-H, and 121°C-H treatments (Group II) gradually returned to the level of CCS-H (Fig. 3F).

### Correlation between α-/β-Type Microbes and the Incidence of Disease

**Fig. 4:**
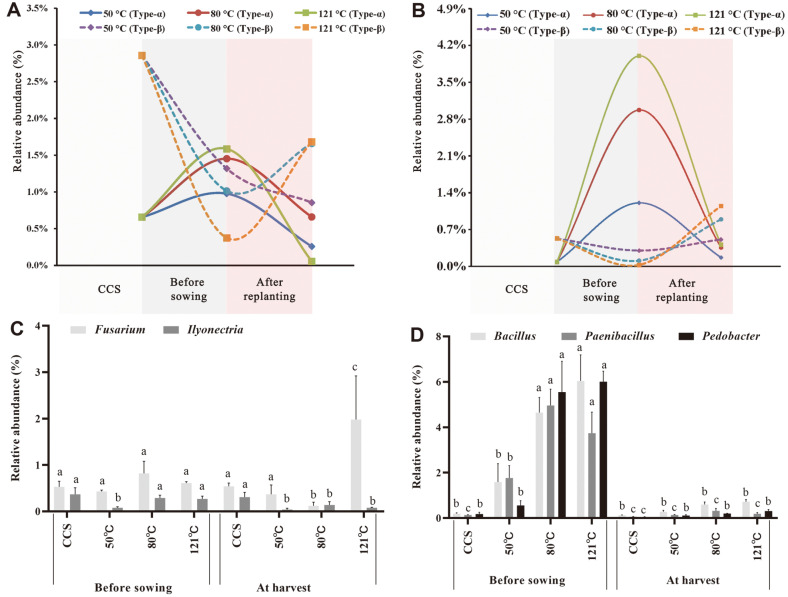
Changes in the relative abundance of the α-/β-type microbes after heating at 50, 80, and 121°C. (**A**) Change in the fungal community in consecutively cultivated soil (CCS). (**B**) Change in the bacterial community in CCS. (**C**) The abundance of sanqi root rot pathogens. (**D**) The abundance of antagonistic bacteria. The CCS was a control. *n* = 3 for the control/treatment group. The data are presented as mean ± SD and were compared using the least significant difference (LSD) test (*p* < 0.05). The α- and β-type microbes are represented by solid and dashed lines, respectively.

For the high-throughput sequencing analysis, we defined the bacteria or fungi that were positively correlated with seedling survival and negatively correlated with disease incidence as α-type microbes, and those that were negatively correlated with seedling survival and positively correlated with disease incidence as β-type microbes. The α-type bacteria, including *Geobacillus*, *Paenibacillus*, *Fictibacillu*, *Cohnell*, *Psychrobacillus*, *Micromonospora*, Polaromonas, and *Brevundimonas* were positively correlated with seedling survival (*p* < 0.05); *Sporosarcina*, *Bacillus*, *Pedobacter*, *Paenibacillus*, *Fictibacillus*, *Cohnella*, *Paenisporosarcina*, *Mucilaginibacter*, *Psychrobacillus*, *Planomicrobium*, and *Dyadobacter* were negatively correlated with disease incidence (*p* < 0.01). The β-type bacteria, including *Acidibacter*, *Bryobacter*, and *Marinicella* were negatively correlated with seedling survival (*p* < 0.01); *Arenimonas*, *Gemmatimonas*, *Bryobacter*, *Marinicella*, *Opitutus*, *Haliangium*, and *Phenylobacterium* were positively correlated with disease incidence (*p* < 0.01) (Table S1). The α-type fungi, such as *Zopfiella*, were positively correlated with seedling survival (*p* < 0.01), whereas *Alternaria*, *Circinotrichum*, *Lophodermium*, *Pseudogymnoascus*, *Zopfiella*, and *Sympodiomyces* were negatively correlated with disease incidence (*p* < 0.05). The β-type fungi, including *Mortierella* and *Coprinellus*, were negatively correlated with seedling survival (*p* < 0.01), whereas *Coprinellus* and *Coprinellus* were positively correlated with disease incidence (*p* < 0.05) (Table S2). After the CCS was heated, the relative abundance of most α-type microbes increased significantly, while the relative abundance of the β-type microbes decreased significantly. Notably, the α- and β-type microbes recovered gradually to the CCS level at harvest (Figs. 4A and 4B). Furthermore, the composition of sanqi root rot pathogens, such as *Fusarium* and *Ilyonectria*, as well as antagonistic bacteria, including *Bacillus*, *Paenibacillus*, and *Pedobacter*, were further analyzed. The results showed that the heat treatment before sowing only slightly affected the abundance of sanqi root rot pathogens, regardless of the temperature (50, 80, or 121°C). In contrast, the relative abundance of *Fusarium* increased significantly (*p* < 0.05) at harvest in CCS treated at 121°C (Fig. 4C). The abundance of antagonistic bacteria increased significantly compared to that in CCS (*p* < 0.05), particularly in the 80°C and 121°C groups. Notably, the abundance of antagonistic bacteria returned to the CCS level in all treatment groups at harvest (Fig. 4D). These results provide crucial evidence that the soil heat treatments increased the relative abundance of beneficial bacteria; however, relative abundance gradually decreased at replanting, which may be a key factor in the negative feedback between sanqi and the soil.

### Isolation of α-Type Bacteria and Its Antagonistic Effect In Vitro

**Fig. 5:**
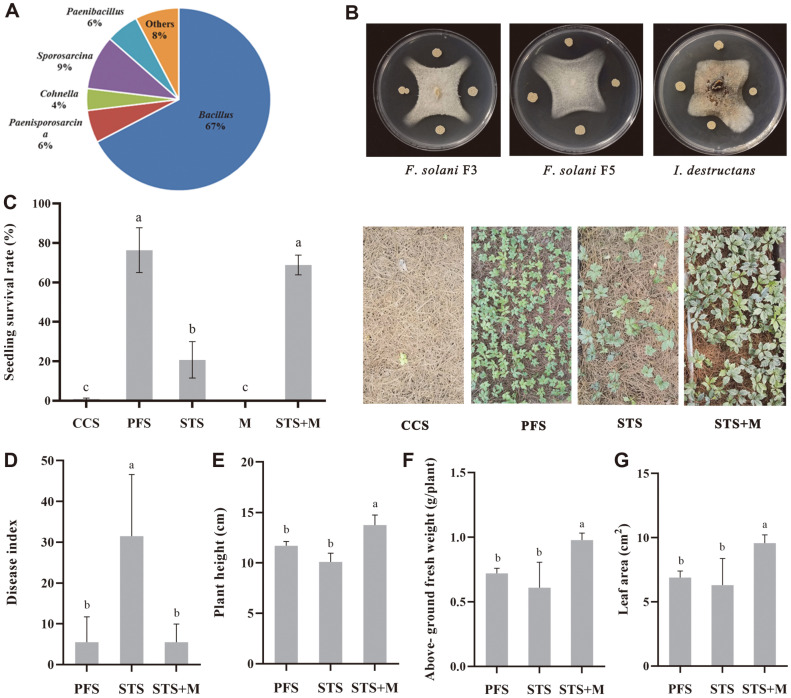
Verification of the ecological function of α-type bacteria. (**A**) Statistical analysis of the surviving bacteria in the CCS after streaming at 80°C. (**B**) Antagonistic activity of *Bacillus* M against sanqi root rot pathogens. (C–G) Effect of STS combined with *Bacillus* M amendment on sanqi seedling survival, the occurrence of wilt, and growth. Untreated CCS and uncultivated PFS were used as controls. *n* = 4 for the control/treatment group. The data are presented as mean ± standard deviation (SD) and were compared using Fisher’s least significant difference (LSD) test (*p* < 0.05).

To further explore the effect of bacterial community structure on sanqi growth, heat-tolerant bacteria that survived in the CCS were isolated after 30 min at 80°C. As a result, 52 bacterial isolates were obtained; 48 were α-type bacteria, including *Bacillus*, *Sporosarcina*, *Paenibacillus*, *Paenisporosarcina*, and *Cohnella*, and the isolation frequencies were 67%, 9%, 6%, 6%, and 4%, respectively (Fig. 5A). The *Bacillus* M isolate was selected in a dual culture test, which showed stronger inhibition of sanqi root rot pathogens, such as *F. solani* (strains F3 and F5) and *I. destructans* DS006 (Fig. 5B).

### Steaming Combined with a *Bacillus* Amendment Increases Seedling Survival and Suppresses Soil-Borne Disease in the Field

As shown in Fig. 5C, the seedling survival rate in the CCS control plots was zero, 76.0% for PFS, 21.0% for STS, and zero for the *Bacillus* M amendment alone. STS combined with *Bacillus* M significantly increased the seedling survival rate to 69%, which was similar to that in the PFS (*p* < 0.05). Additionally, STS combined with *Bacillus* M significantly reduced the incidence of seedling wilt by 86.5% compared to STS (Fig. 5D). Plant height, above-ground fresh weight, and leaf area increased ~36.1%, 52%, and 60.6%, respectively, relative to STS (Figs. 5E–5G). Taken together, these results indicate that NPSF of *P. notoginseng* was dominated by a change in α-type bacteria.

## Discussion

Although previous studies have reported that steaming eliminates NPSF for sanqi cultivation [26, 32], the micro-ecological mechanism remains unknown. In the present study, we explored the microbial community after soil steaming through 16S rRNA and ITS sequencing techniques. We found that the appropriate soil heat treatment of 80°C was more conducive for seedling growth on CCS than the high (121°C) and low (50°C) temperature treatments. Furthermore, steaming promotes growth and disease suppression of *Panax notoginseng* by interfering with the bacterial community rather than the fungi in the consecutively cultivated system. Importantly, steaming increased the α-type microbes and decreased β-type relative abundances before sowing, however, richness and diversity gradually recover to the level of CCS, regardless of α- or β-type microbes, after replanting. Thus, the amendment of α-type bacteria (such as *Bacillus* spp.) was critical after steaming.

Soil conditions should be improved before replanting, particularly for sanqi production, to maintain long-term soil health and sustainable agriculture [18, 19, 26, 33]. Consistent with previous studies, heat treatment increased sanqi seedling survival and reduced the occurrence of wilt compared to CCS. The 80°C treatment resulted in higher emergence and survival rates than those observed at 50 and 121°C (Fig. 1). One possible reason is that the 121°C heat treatment resulted in a serious imbalance in the microbial community, regardless of α- or β-type microbes, likely to death or transition to dormancy [25]. For example, *Fusarium* spp. can develop chlamydospores and survive at high temperature, however, they were not detected above 80°C heat treatment (Fig. 2). Considering the selectivity and sensitivity of the plate separation method, the specific medium may be better for detecting some microorganisms with dormant structure. In contrast, the 50°C heat treatment is not enough to eliminate pathogenic microorganisms in the soil, and they easily recolonize once replanted.

Successful colonization and an adequate microbial population are key factors in biological management [34, 35]. The present study suggests although the steam treatment decreased the Chao1 index of fungal community, it did not easily recolonize during replanting (Fig. 3A), while as well as the steam treatment and replanting did not affect soil fungal diversity (Fig. 3B). Even the major pathogenic agents, such as *Fusarium* spp. and *Ilyonectria* spp., were maintained at relatively low levels compared to before steaming, and in consecutively cultivated soil (Fig. 4C). Consistent with previous studies, steaming changed the abundance of bacteria [18, 33]. In our study, steaming reduced the Chao1 and Shannon’s diversity indices for bacteria with increasing steam temperature; however, richness and diversity gradually increased to the level of CCS after replanting (Figs. 3C and 3D). As *Bacillus* and *Paenibacillus* species produce resistant endospores which allow them to survive harsh conditions such as heat and acid stress [33, 36], the present study also showed that the relative abundances of most of the α-type microbes significantly increased after soil steaming (Figs. 4A and 4B), and particularly the α-type bacteria, such as *Bacillus*, *Paenibacillus*, and *Pedobacter*, were retained (Fig. 4D). Thus, the NPSF of *P. notoginseng* may be dominated by a change in the community structure of α-type bacteria rather than fungi. In addition, most of the heat-tolerant bacteria have been designated as plant growth-promoting rhizobacteria (PGPR), and have been successfully used for plant disease management for many years, via both direct and indirect mechanisms [37, 38].

Steaming had a short-lasting environmental impact on the soil [18]. Several studies and reviews have postulated that plants actively recruit beneficial soil microorganisms via specific constituents in root exudates, to colonize the rhizosphere and counteract NPSF [24, 33, 39]. Although steam increased the relative abundances of most of the α-type microbes before sowing, richness and diversity gradually recover to the level of CCS, regardless of whether they are α- or β-type microbes, after replanting (Figs. 4A and 4B), and even for PGPR (Fig. 4D). On the one hand, while harmful microbes represent a continuing obstacle for *P. notoginseng*, on the other hand, steaming combined with *Bacillus* amendment may provide long-term protection.

## Supplemental Materials

Supplementary data for this paper are available on-line only at http://jmb.or.kr.
