# Supplementary material for: Appropriate Soil Heat Treatment Promotes Growth and Disease Suppression of Panax notoginseng by Interfering with the Bacterial Community
Source: J Microbiol Biotechnol. 2022 Feb 17;32(3):294–301. doi: 10.4014/jmb.2112.12005 (PMC9628859; doi:10.4014/jmb.2112.12005)
Supplement: Supplementary file 1 [file jmb-32-3-294-supple.pdf]

J. Microbiol. Biotechnol.
https://doi.org/10.4014/jmb.2112.12005

Supplemental Materials

Table S1 Correlation analysis of the relative abundance of α-/β-type bacteria and seedling survival and disease incidence.

Type	Genus level	Person correlation		Relative abundance			
		Survival rate	Incidence rate	CCS	50°C	80°C	121°C
α	*Sporosarcina*	0.205	-0.620 **	0.0026 c	0.0390 c	0.1977 b	0.3448 a
	Massilia	0.165	-0.467 *	0.0034 b	0.0391 ab	0.0376 ab	0.1017 a
	Geobacillus	0.417 *	-0.350	0.0006 a	0.0541 a	0.0358 a	0.0020 a
	Bacillus	0.325	-0.631 **	0.0020 b	0.0159 b	0.0465 a	0.0604 a
	Pedobacter	0.285	-0.609 **	0.0018 b	0.0056 b	0.0555 a	0.0601 a
	Paenibacillus	0.435 *	-0.676 **	0.0014 c	0.0177 bc	0.0496 a	0.0374 ab
	Fictibacillus	0.502 *	-0.627 **	0.0002 b	0.0171 ab	0.0361 a	0.022 ab
	Cohnella	0.441 *	-0.682 **	0.0004 b	0.0087 b	0.0255 a	0.0174 a
	Paenisporosarcina	0.19	-0.582 **	0.0001 c	0.0031 c	0.0089 b	0.0185 a
	Mucilaginibacter	0.068	-0.529 **	0.0002 c	0.0006 c	0.0052 b	0.0156 a
	Psychrobacillus	0.500 *	-0.635 **	0.0004 c	0.0041 bc	0.0131 a	0.0067 b
	Micromonospora	0.445 *	0.074	0.0010 ab	0.0034 a	0.0017 ab	0.0002 b
	Planomicrobium	0.134	-0.517 **	0.0001 b	0.0008 b	0.0031 ab	0.0068 a
	Herbaspirillum	0.200	-0.464 *	0.0001 b	0.0019 ab	0.0022 ab	0.0059 a
	Polaromonas	0.468 *	-0.089	0.0001 b	0.0013 ab	0.0053 a	0.0019 ab
	Brevundimonas	0.413 *	-0.037	0.0009 b	0.0028 ab	0.0054 a	0.0054 a
	Dyadobacter	0.205	-0.563 **	0.0001 b	0.0006 b	0.0042 a	0.006 a
	Noviherbaspirillum	0.173	-0.493 *	0.0001 b	0.0016 b	0.0016 b	0.0073 a
β	*Arenimonas*	0.020	0.583 **	0.0074 a	0.0032 b	0.0006 c	0.0001 c
	Gemmatimonas	0.037	0.527 **	0.0034 a	0.0026 a	0.0009 b	0.0000 b
	Acidibacter	-0.636 **	0.443 *	0.0111 a	0.0061 b	0.0019 c	0.0001 d
	Devosia	0.200	0.455 *	0.0030 a	0.0033 a	0.0025 ab	0.0014 b
	Bryobacter	-0.553 **	0.814 **	0.0064 a	0.0032 b	0.0015 c	0.0002 d
	Marinicella	-0.772 **	0.767 **	0.0042 a	0.0004 b	0.0000 c	0.0000 c
	Planctomyces	0.053	0.469 *	0.0043 a	0.0046 a	0.0013 b	0.0001 b
	Opitutus	-0.054	0.635 **	0.0038 a	0.0014 b	0.0005 c	0.0001 d
	Haliangium	-0.238	0.59 4**	0.0073 a	0.0031 b	0.0006 c	0.0001 c

Type	Genus level	Person correlation		Relative abundance			
		Survival rate	Incidence rate	CCS	50°C	80°C	121°C
	Phenylobacterium	0.159	0.517 **	0.002 ab	0.0022 a	0.0010 ab	0.0007 b

Lowercase letters $p < 0.05$, uppercase letters $p < 0.01$. $^{*}p < 0.05$ and $^{**}p < 0.01$.

Table S2 Correlation analysis of the relative abundance of α-/β-type fungi and seedling survival and disease incidence.

Type	Genus level	Person correlation		Relative abundance			
		Survival rate	Incidence rate	CCS	50°C	80°C	121°C
α	*Alternaria*	0.042	-0.455 *	0.0002 b	0.0027 b	0.0068 b	0.0343 a
	Circinotrichum	0.272	-0.496 *	0.0002 a	0.0010 a	0.0045 a	0.0040 a
	Lophodermium	0.041	-0.456 *	0.0000 b	0.0005 b	0.0013 b	0.0052 a
	Pseudogymnoascus	0.313	-0.607 **	0.0371 a	0.0508 a	0.0578 a	0.0476 a
	Zopfiella	0.576 **	-0.409 *	0.0014 b	0.0031 b	0.0097 a	0.0015 b
	Sympodiomyces	0.337	-0.441 *	0.0005 a	0.0005 a	0.0071 a	0.0026 a
β	*Mortierella*	-0.626 **	0.387	0.0951 a	0.0382 b	0.0183 b	0.0060 b
	Coprinellus	-0.517 **	0.416 *	0.0090 a	0.0000 b	0.0019 ab	0.0000 b
	Monographella	0.226	0.456 *	0.0056 b	0.0101 ab	0.0144 a	0.0041 b
	Cryptococcus	-0.440 *	0.177	0.0046 a	0.0044 a	0.0060 a	0.0049 a

Lowercase letters $p < 0.05$, uppercase letters $p < 0.01$. $^{*}p < 0.05$ and $^{**}p < 0.01$.
